# The Comprehensive Toxicological Assessment of Total Chromium Impurities in Traditional Herbal Medicinal Product with Thymi herba (*Thymus vulgaris* L. and *Thymus zygis* L.) Available in Pharmacies in Poland: Short Communication

**DOI:** 10.1007/s12011-021-02864-2

**Published:** 2021-08-10

**Authors:** Kamil Jurowski, Maria Fołta, Barbara Tatar, Mirosław Krośniak

**Affiliations:** 1 Institute of Medical Studies, Medical College, Rzeszów University, Al. mjr. W. Kopisto 2a, 35-959 Rzeszów, Poland; 2 Department of Food Chemistry and Nutrition, Medical College, Jagiellonian University, Medyczna 9, 30-688 Kraków, Poland

**Keywords:** *Thymus vulgaris* L., Cr, Elemental impurities, ICH Q3D, Toxicology, Total chromium, Traditional herbal medicinal products, Herbal drugs

## Abstract

Scientific reports about elemental impurities in final pharmaceutical products are essential from a regulatory point of view; unfortunately, there is a lack of studies about this important toxicological topic. The aim of our short communication was determination of total Cr impurities in traditional herbal medicinal products (THMP) with Thymi herba (*Thymus vulgaris* L. and *Thymus zygis* L.) available in Polish pharmacies (*n* = 6, because only six manufacturers produce this kind of pharmaceutical products in Poland). The total content of Cr impurities was determined by atomic absorption spectrometry using electrothermal atomization (ETAAS). Applied comprehensive toxicological risk assessment was based on three main tiers: Tier 1, the comparison of raw results as total Cr impurities profile (metal per L of THMP) with ICH Q3D guideline standards; Tier 2, the estimation of total Cr exposure with a single dose; and Tier 3, the estimation of total Cr daily exposure. We confirmed that total Cr impurities were present in all analyzed THMP with thymi herba (the observed level was below 6.0 µg/L). Total Cr concentration in a single dose can be deceptively high in comparison to the raw results but is not a threat to patients (20.15–63.45 ng/single dose). Moreover, the estimation of total Cr daily exposure shows that all analyzed THMP are characterized by daily dose (40.30–181.41 ng/day) below PDE value (10,700 mg/day); hence, all products meet the standards of ICH Q3D elemental impurities guideline.

## Introduction

Thyme is the common name for the leaves and flowers of the plants *Thymus vulgaris* L. or *Thymus zygis* L, usually defining together as *Thymi herba* as a single substance [1]. Mentioned ingredients are applied as active pharmaceutical ingredients (API) in traditional herbal medicinal products (THMP) applied in productive cough associated with cold [1, 2]. The herbal preparation, for example, in Poland is based on liquid extract (DER 1:2–2.5) extraction solvent mixture of ammonia 10% (1 part), glycerol 85% (20 parts), ethanol 90% v/v (70 parts), and purified water (109 parts) [2]. It should be emphasized that *Thymi herba* can be a potential source of elemental impurities (EIs). For example, the total Cr content of the thyme (*Thymi vulgaris herba*, Bionit Ltd., Kistarcsa, Hungary) measured by Kovács et al. [3] was 12,05 l g/100 g, and the value published by Fischer [4] was 10,00 l g/100 g. On the other hand, value reported by Garcia et al. [5] was 85 l g/100 g. The elemental content of thyme depends mainly on the growing location because it can accumulate large quantities of elements. Based on preliminary studies (*n* = 3) described by Szentmihályi et al. [6], the EIs of drug components and Species thymi composita (mg/kg dry weight) are quite different. For example, Cr level was relatively high (11.51 ± 0.24 mg/kg) [6] in comparison to other elemental impurities. It should be underlined that Cr is an “enigmatic” element. Currently, this element can only be considered pharmacologically active and not an essential element [7]. This element has a number of oxidation states; especially Cr(III) and Cr(VI) are most important. There is no doubt that Cr(III) is the most abundant form in the environment and is an essential element that plays a role in glucose metabolism [7]. The deficiency of this Cr form causes changes in the metabolism of glucose and lipids and may be associated with maturity-onset diabetes, cardiovascular diseases, and nervous system disorders [8]. Additionally, soluble compounds of this Cr form are poorly toxic; e.g., LD_50_ (rodent, oral) was in the range 100–400 mg/kg BW. On the other hand, compounds with Cr(VI) are manufactured and hence are not present in the environment. However, this form of Cr is genotoxic (animal studies—inhalation), which was confirmed by human epidemiological studies—carcinogenic in the respiratory tract [9]. Moreover, any amount of Cr(VI) entering the cells can initiate tumor formation [10]. Interesting are evidence that Cr(VI) can be reduced to Cr(III) in the gastrointestinal tract. So, only intakes that exceed the reducing capacity of the stomach will result insignificant absorption of Cr(VI) across the gastrointestinal mucosa [9]. Regardless of chemical form, usually Cr impurities in pharmaceuticals are usually determined as total Cr level.

The aim of our short article was the determination of Cr impurities in THMP with Thymi herba (*Thymus vulgaris* L. and *Thymus zygis* L.) available in Polish pharmacies. We investigated all available products (*n* = 6) in Poland. The total content of Cr impurities was determined by atomic absorption spectrometry using electrothermal atomization (ETAAS). To the best of our knowledge, the Cr levels in THMP with Thymi herba available in European pharmacies are reported here for the first time.

## Materials and Methods

### Chemicals

All chemicals and metal stock standard solutions were obtained from Merck (Darmstadt, Germany). Applied solutions were prepared with ultrapure demineralized water (Milli-Q water purification system, Millipore, Bedford, MA, USA). Working solutions of Cr (0.0, 12.5, 20.0, 50.0, and 100.0 μg/L) were prepared from the stock solutions of 1-mg/mL chromium(III) nitrate (CertiPUR®) applying demineralized water (mentioned earlier) in 0.5-mol/L nitric acid. The certified reference material (BCR-482; IRMM, Belgium) was material prepared from lichen. The purge gas was argon at 5 N purity.

### Description of Investigated Samples

All available THMP with Thymi herba in Polish pharmacies (*n* = 6) were considered. All products were purchased from pharmacies in the Malopolska region (Kraków) and Podkarpacie region (Rzeszów). All pharmaceutical products were coded: A–F, the double-blind approach was applied. Most of the samples (*n* = 4/6) were over-the-counter (OTC) drugs. The summary of analyzed samples is described briefly in Table 1. Before the analysis, purchased products were stored at a temperature of 20 °C. Additional treatment of samples (like homogenization and digestion) was not necessary, because all HMP were liquid samples (Thymi herba drops). Hence, in situ analysis was applied at measurement step.

**Table 1:** The summary of analyzed THMP with Thymi herba (*Thymus vulgaris* L. and *Thymus zygis* L.) available in Polish pharmacies

Sample	Posology	License	Lot number	Type of product
A	Oral use: 10 mL 3 times daily	NA	10,420	DS
B	Oral use: 10–15 mL 2 times daily	IL-4528/LN	11,020	OTC
C	Oral use: 10 mL 3 times daily	IL-3695/LN	11,020	OTC
D	Oral use: 10–15 mL 2 times daily	IL-0995/LN	20,520	OTC
E	Oral use: 10–15 mL 2 times daily	R/0460	40,320	OTC
F	Oral use: 10 mL 3 times daily	NA	01AF0820	DS

*DS*, dietary supplement; *NA*, not applicable; *OTC*, over-the-counter drug

### The Designed Procedure of Assessment of Total Cr Impurities in THMP with Thymi herba

The appropriate procedure of assessment of total Cr impurities in THMP with Thymi herba was designed. The comprehensive toxicological risk assessment was based on three main tiers:

- Tier 1: The comparison of raw results as total Cr impurities profile (metal per L of THMP) with ICH Q3D guideline standards
- Tier 2: The estimation of total Cr exposure with a single dose (based on posology—Table 1)
- Tier 3: The estimation of total Cr daily exposure based on step 2 and posology (Table 1) and comparison with PDE (permitted daily exposure) values suggested by ICH Q3D guideline

### Total Cr Determination and Data Processing

The determination of total Cr in the analyzed samples was conducted by PerkinElmer 5100 ZL atomic absorption spectrometer (PerkinElmer, Norwalk, CT, USA) with Zeeman background correction and with electrothermal atomization (ETAAS technique). The emission source was Cr hollow cathode lamp (wavelength = 357.9 nm; intensity = 0.5 mA). The special time–temperature program for Cr determination was applied—Table 2. The sample volume was 40 μL, and for signal evaluation, integrated absorbance (peak area) was applied.

**Table 2:** The special time–temperature program for Cr determination

Condition(s)	Value
Step 1, °C	110
Ramp/hold, s	5/15
Step 2, °C	180
Ramp/hold, s	30/10
Step 3, °C	450
Ramp/hold, s	1/5
Step 4, °C	500
Ramp/hold, s	5/5
Step 5, °C	1500
Ramp/hold, s	15/30
Step 6, °C	2450
Ramp/hold, s	0/5
Step 7, °C	2500
Ramp/hold, s	½
L’vov platform	Yes
Integration time, s	4
Injected: sample volume, µL	40.0

The analytical calibration strategy was applied including working solutions of Cr (5.0; 10; 50.0, 100.0, and 150.0 μg/L) prepared from the stock solutions of 1000 μg/mL (chromium(III) nitrate; CertiPUR®) using ultrapure demineralized water in 0.5-mol/L nitric acid. The calibration function indicated an acceptable correlation coefficient (*R* = 0.9989). Hence, there was good linearity of instrumental response with metal concentrations.

For quality control, the certified reference material was applied (lichen; BCR-482 IRMM, Belgium); the certified value for Cr was 0.134 mg/kg, and the measured value was 0.137 mg/kg. Hence the recovery was 97.81%. The LOD was 1.65 μg/L, and LOQ was 4.95 μg/L.

The confirmation of our methodology were articles published earlier [11–14].

All data were analyzed using statistical software Origin 2021 Pro, the ultimate software for graphing and analysis (OriginLab Corporation, One Roundhouse Plaza, Suite 303, Northampton, MA 01,060, USA) licensed by the Jagiellonian University in Krakow. The resultant data of five independent replicates (five replicate samples from one bottle of each product) were expressed as the mean ± standard deviation.

### Applied Toxicological Risk Assessment Approach

As was mentioned earlier, the appropriate procedure of assessment of total Cr impurities in THMP with Thymi herba was designed. The last step of our process was a comprehensive toxicological risk assessment which consists of three main tiers:

- Tier 1: The comparison of raw results as total Cr impurities profile (metal per L of THMP) with ICH Q3D guideline standards [15].
- Tier 2: The estimation of total Cr exposure with a single dose; i.e., the worst-case scenario was applied for estimation of maximum Cr exposure with a single dose; based on the highest values of posology (Table 1), the levels of total Cr to which the patient is exposed for a single dose of the THMP with thymi herba were estimated (Table 3).
- Tier 3: The estimation of total Cr daily exposure based on Tier 2, posology (Table 1) in comparison with oral PDE value (10,700 µg/day) suggested by ICH Q3D guideline (Table 4).

**Table 3:** The estimation of total Cr exposure for a single dose of each THMP with thymi herba available in Polish pharmacies

Traditional herbal medicinal product with thymi herba	The estimation of total Cr exposure with a single dose	
	ng/single dose	SD
A	23.02	1.45
B	63.45	0.89
C	24.12	0.75
D	60.06	0.95
E	20.15	0.62
F	60.47	1.45

*SD* standard deviation

**Table 4:** The estimation of total Cr exposure for a daily dose of each THMP with thymi herba available in Polish pharmacies

Traditional herbal medicinal product with thymi herba	The estimation of total Cr exposure for a daily dose	
	ng/daily dose	SD
A	69.06	1.45
B	126.90	0.89
C	72.36	0.75
D	120.12	0.95
E	40.30	0.62
F	181.41	1.45

*SD* standard deviation

## Results and Discussion

### Tier 1: Total Cr Impurities Profile

**Fig. 1:**
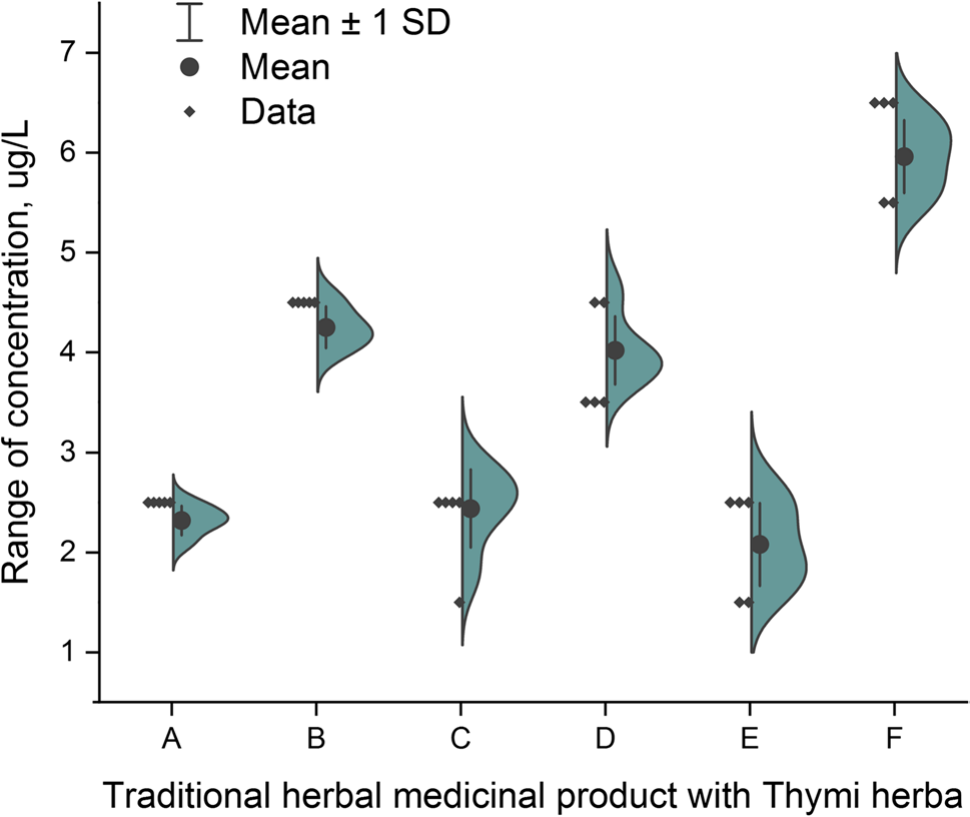
The total Cr content (impurities profile) as half violin plot for total Cr content in all analyzed traditional herbal medicinal products (A–F) with thymi herba available in Polish pharmacies

The total Cr content (impurities profile) in all analyzed THMP is shown as the half violin plot in Fig. 1.

The total Cr impurities profile (Fig. 1) shows that Cr impurities were present in all analyzed THMP with thymi herba (in the range: 2.0 µg/L–6.0 µg/L). However, the observed level was below 6.0 µg/L. The lowest level was similar in three samples:

E (2.0 ± 0.3 µg/L), A (2.3 ± 0.2 µg/L), and C (2.4 ± 0.3 µg/L). On the other hand, the highest level was in sample F (6.0 ± 0.4 µg/L). Based on pilot studies (*n* = 3) described by Szentmihályi et al. [3], Cr level was relatively high (11.51 ± 0.24 mg/kg), which is not in accordance with our studies. Considering acceptable limits for impurities, including all Cr forms in pharmaceuticals via the oral route, recommended by the ICH Q3D guideline (1100 µg/g [12]), all of the analyzed products meet the requirements by the ICH Q3D guideline.

The simple descriptive statistics show that the range of the total Cr impurities was 4.0 µg/L and the ratio of the maximum value to the minimum value was 3:1. Additionally, skewness (1.15) and kurtosis (1.85) confirm the distribution of results and their consistency. The half violin plot (Fig. 1) confirms the symmetric distribution of the data and illustrated range of the total Cr concentration in analyzed THMP with thymi herba.

### Tier 2: The Estimation of Total Cr Exposure with a Single Dose

For the final assessment of total Cr exposure for human health (in daily intake of applied THMP), the required second step is to estimate Cr exposure with a single dose. It is not easy because there are many ways to use these products based on posology and method of administration described in community herbal monograph on *Thymus vulgaris* L. and *Thymus zygis* L., herba by the European Medicines Agency [1, 2]. Due to the toxicological risk assessment, the worst-case scenario was applied to estimate Cr exposure with a single dose. Hence, based on the highest values of posology described earlier (see Table 1), the levels of total Cr to which the patient is exposed for a single dose of the THMP with thymi herba is shown in Table 3.

The performed estimation in single dose shows that due to differences in posology, exposure in a single dose can be relatively high compared to that in the earlier raw results.

### Tier 3: The Estimation of Total Cr Daily Exposure

The final step in the conducted toxicological risk assessment of Cr impurities is the estimation of total Cr daily exposure based on the last step (Tier 2) and posology (Table 1) in comparison with oral PDE value (10,700 µg/day) suggested by ICH Q3D guideline [15]. The estimated values of total Cr impurities in daily exposure are shown in Table 4.

The estimated results for daily exposure to Cr are variable (40.30–181.41 ng/day), but on relatively low level (< 200 ng/day). The most relevant safety information for Cr in pharmaceuticals were obtained from the second year National Toxicology Program studies [16] on the carcinogenicity of Cr(III) picolinate administered in feed to rats and mice at 2000, 10,000, and 50,000 ppm. The established NOAEL was the low dose of 90-mg/kg Cr(III) picolinate (10.7 mg/kg/day Cr(III)) in rats based on increase in the incidence of preputial gland adenoma in male rats at 460 mg/kg [13]. Hence, the calculated PDE value for Cr was 10,700 mg/day [12]. The results of our estimation of Cr exposure for a daily dose show that all analyzed THMP are characterized by results extremely below PDE value (the highest result was 181.41 ng/day).

## Conclusions and Recommendations

This work confirmed that total Cr impurities were present in all analyzed THMP with thymi herba (the observed level was below 6.0 µg/L). Total Cr concentration in a single dose (20.15–63.45 ng/single dose) can be deceptively high in comparison to the raw results but is not a threat to patients. Moreover, the estimation of total Cr daily exposure (40.30–181.41 ng/day) shows that all analyzed THMP are characterized by results extremely below PDE value (10,700 mg/day); hence, all products meet the standards of ICH Q3D guideline. It can be summarized that the proposed toxicological risk assessment approach was well designed and confirm that all analyzed THMP with Thymi herba (*Thymus vulgaris* L. and *Thymus zygis* L.) available in pharmacies in Poland are safe due to total Cr impurities. Additional studies about other EI in this kind of THMP available in Europe are desired.

## Data Availability

All data generated or analyzed during this study are included in this published article and its supplementary information file.

## Abbreviations

AAS: Atomic absorption spectrometry technique
EI: Elemental impurities
ETAAS: Electrothermal atomization atomic absorption spectrometry
THMP: Traditional herbal medicinal product
ICH Q3D: International Council for Harmonization of Technical Requirements for Pharmaceuticals for Human Use
PDE: Permitted daily exposure
TRA: Toxicological risk assessment
SD: Standard deviation

## References

[1] EMA/HMPC/342332/2013, Committee on Herbal Medicinal Products (HMPC), Community herbal monograph on Thymus vulgaris L. and Thymus zygis L., herb, Available 26.06.2021 at https://www.ema.europa.eu/en/documents/herbal-monograph/final-community-herbal-monograph-thymus-vulgaris-l-thymus-zygis-l-herba_en.pdf. Accessed 26 June 2021

[2] EMA/HMPC/342334/2013, Committee on Herbal Medicinal Products (HMPC), Assessment report on Thymus vulgaris L., vulgaris zygis L., herba, Available 26.06.2021 at https://www.ema.europa.eu/en/documents/herbal-report/final-assessment-report-thymus-vulgaris-l-vulgaris-zygis-l-herba_en.pdf. Accessed 26 June 2021

[3] Kovács R, Béni Á, Karosi R, Sógor C, Posta J (2007). Investigation of chromium content in foodstuffs and nutrition supplements by GFAAS and determination of changing Cr(III) to Cr(VI) during baking and toasting bread. Food Chem.

[4] Fischer JA (1990). The chromium program.

[5] Garcia M, Cabrera C (2000). Chromium levels in spices and aromatic herbs. Sci Environ.

[6] Szentmihályia K, Maya Z, Then M, Hajdú M, Böszörményi A, Balázs A, Szőke É (2012). Metal elements, organic agents in a herbal remedy, species thymi composita, and its drug constituents. Eur Chem Bull.

[7] Vincent JB (2017). New evidence against chromium as an essential trace element. J Nutr.

[8] Anderson RA (1993). Recent advances in the clinical and biochemical effects of chromium deficiency. Prog Clin Biol Res.

[9] Anderson RA (1995). Chromium and parenteral nutrition. Nutrition.

[10] Sun H, Brocato J, Costa M (2015). Oral chromium exposure and toxicity. Curr Environ Health Rep.

[11] Jurowski K, Fołta M, Tatar B, Berkoz M, Krośniak M (2021) Ni and Cr impurities profile in Valeriana officinalis L., radix-based herbal medicinal product available in Polish pharmacies due to ICH Q3D guideline. Reg Tox Phar. 10.1016/j.yrtph.2021.104945 (Online ahead of print) 10.1016/j.yrtph.2021.104945 33991634

[12] Jurowski K, Krośniak M, Fołta M, Tatar B, Cole M, Piekoszewski W (2019). Safety assessment of the trace element impurities Ni and Cr in pharmaceutical herbal products for teething from Polish pharmacies. Biol Trace Elem Res.

[13] Jurowski K, Krośniak M, Fołta M, Cole M, Piekoszewski W (2019). The toxicological analysis of Cu, Mn and Zn as elemental impurities in pharmaceutical herbal products for teething available in pharmacies in Poland. J Trace Elem Med Biol.

[14] Jurowski K, Krośniak M, Fołta M, Cole M, Piekoszewski W (2019). The analysis of Cu, Mn and Zn content in prescription food for special medical purposes and modified milk products for newborns and infants available in Polish pharmacies from toxicological and nutritional point of view. J Trace Elem Med Biol.

[15] ICH guideline Q3D (R1) on elemental impurities. 28 March 2019 EMA/CHMP/ICH/353369/2013 Committee for Human Medicinal Products. https://www.ema.europa.eu/en/ich-q3d-elementalimpurities. Accessed 26 June 2021

[16] National Toxicology Program. Technical report on the toxicology and carcinogenesis studies of chromium picolinate monohydrate (CAS NO. 27882–76–4) in F344/N rats and B6C3F1 mice (feed studies). National Toxicology Program, Public Health Service ,U.S. Department of Health and Human Services. 2010;NTPTR556 20725156

